# Application of the Initial Rate Method in Anaerobic Digestion of Kitchen Waste

**DOI:** 10.1155/2017/3808521

**Published:** 2017-05-04

**Authors:** Lei Feng, Yuan Gao, Wei Kou, Xianming Lang, Yiwei Liu, Rundong Li, Meiling Yu, Lijie Shao, Xiaoming Wang

**Affiliations:** ^1^Liaoning Province Clean Energy Key Laboratory, Shenyang Aerospace University, Shenyang Daoyi Street 37, Shenyang 110136, China; ^2^Liaoning Institute of Energy Resources, 65# Yingquan St., Yingkou, Liaoning, China; ^3^Liaoning Academy of Environmental Sciences, 30# Shuangyuan St., Shenyang, Liaoning 115003, China

## Abstract

This article proposes a methane production approach through sequenced anaerobic digestion of kitchen waste, determines the hydrolysis constants and reaction orders at both low total solid (TS) concentrations and high TS concentrations using the initial rate method, and examines the population growth model and first-order hydrolysis model. The findings indicate that the first-order hydrolysis model better reflects the kinetic process of gas production. During the experiment, all the influential factors of anaerobic fermentation retained their optimal values. The hydrolysis constants and reaction orders at low TS concentrations are then employed to demonstrate that the first-order gas production model can describe the kinetics of the gas production process. At low TS concentrations, the hydrolysis constants and reaction orders demonstrated opposite trends, with both stabilizing after 24 days at 0.99 and 1.1252, respectively. At high TS concentrations, the hydrolysis constants and the reaction orders stabilized at 0.98 (after 18 days) and 0.3507 (after 14 days), respectively. Given sufficient reaction time, the hydrolysis involved in anaerobic fermentation of kitchen waste can be regarded as a first-order reaction in terms of reaction kinetics. This study serves as a good reference for future studies regarding the kinetics of anaerobic digestion of kitchen waste.

## 1. Introduction

Kitchen waste constitutes a key part of municipal waste, making up as much as 30% to 50% of municipal solid waste according to the National Environment Bulletin [1]. In China alone in 2012, the amount of kitchen waste produced was 110 million tons [2]. Kitchen waste is sometimes used as animal feed [3, 4], but it is also deposited in landfills, resulting in reduced landfill capacity and environmental issues [5–7]. Consisting of organics containing starch, protein, fiber, and fat, kitchen waste is characterized by high water content, high organic content, and exposure to acidification [8]. Therefore, anaerobic digestion is regarded as an effective way to recycle kitchen waste, as it disposes of the waste without producing contaminants. Meanwhile, methane, a clean energy source, can be produced by anaerobic digestion, making this process an example of good resource utilization [9, 10]. Additionally, solid waste produced by anaerobic digestion contains high nitrogen and phosphorus contents, such that it can be used as organic fertilizer [11, 12] or feed for microalgae that produces biodiesels [13]. In this way, addressing kitchen waste with anaerobic digestion can promote recovery and reuse of resources.

The first step in investigating reaction kinetics is to determine the order of the reaction, which is an indicator of the effect of reactant concentrations on reaction rates, as well as a key parameter for studying the reaction mechanism. Four approaches have so far been proposed for determining reaction order: the integration method, the differential method, the half-life method, and the initial rate method [14, 15]. The initial rate method is an easy and effective method for determining reaction order. Defined as the transient rate at the beginning of a reaction under certain conditions, the initial rate is recognized as a good indicator of the relationship between reactant concentrations and reaction rates as reverse reactions and side reactions are negligible at this stage. Wanasolo et al. determined the hydrolysis constant and reaction order in anaerobic digestion of fruits using the initial rate method [16]. This study investigates trends of the hydrolysis constant and reaction order in anaerobic digestion of kitchen waste during experimental periods based on the initial rate method and the methane yield. The results demonstrate that anaerobic digestion of kitchen waste can be described and predicted by the first-order reaction model.

## 2. Materials and Methods

### 2.1. Raw Materials and Inoculum

Kitchen waste was obtained from the canteen of a local university. Nondegradable substances such as fishbone and disposable chopsticks were removed, and the waste was then cut into 1 cm × 1 cm × 0.5 cm cubes and stored at 4°C. The total solid (TS) concentration and volatile solid (VS) concentration were 23.31% and 92.84%, respectively. Sewage sludge used as inoculum was obtained from a local sewage plant and treated at mild temperatures. The TS concentration, VS concentration, and carbon-to-nitrogen (C/N) ratio of the sewage sludge were 11.26%, 77.79%, and 7.41, respectively.

### 2.2. Equipment and Methods

The customized reactor consisted of three 1 L wide mouth bottles used as a reaction bottle, gas collection bottle, and water collection bottle. For the three low TS concentration tests, 17.8 g, 60.7 g, and 103.6 g raw materials were mixed with 300 mL sludge in the reaction bottle. Water was added as needed so that the solutions in all reaction bottles reached 1 L. In these cases, the initial TS concentrations were 4%, 5%, and 6%, respectively. For the three high TS concentration tests, 330.7 g, 352.1 g, and 373.6 g raw materials were mixed with 150 mL sludge in the reaction bottle. Water was added as needed so that solutions in all reaction bottles reached 500 mL. In these cases, the initial TS concentrations were 19%, 20%, and 21%, respectively. High purity N_2_ was then injected into each reactor for 5 min to eject air. The reaction bottles and gas collection bottles were connected by glass tubes and pretreated latex tubes, followed by sealing using rubber stoppers and sealant. Thermostatic water baths were used to maintain the designated temperature. Each experiment was designed to group 3 parallel samples. After adding water to the fermentation reactor to the level (1L), all reaction bottles were incubated at 37°C in the water bath for 30 d, during which period stirring was conducted twice a day. The pH values of the solutions and gas produced were measured daily to avoid issues such as the inhibition phenomenon.

During the anaerobic fermentation process, all of the influential factors retained their optimal values. Specifically, the fermentation tank was heated in water to maintain an internal temperature of 37°C, which is ideal for anaerobic fermentation. The pH values of the solutions were adjusted to fall within a range of 6.8 to 7.2. In addition, the fermentation tank was shaken twice a day for purposes of stirring, and it was sealed at all times.

### 2.3. Analytical Methods

The products in the TS concentration group and the VS concentration group were heated to 103–105°C and 600°C, respectively. The pH values of the solutions were determined using a digital pH meter. The volume of the produced gas was measured using the saturated salt water replacement method.

### 2.4. Anaerobic Fermentation Kinetic Model

#### 2.4.1. Population Growth Model

The logistic equation is written as follows:

(1) $$\begin{array}{c} P=\frac{P_{max}}{1+\exp\left[\left(4R_{max}\left(\lambda -t\right)/P_{max}\right)+2\right]}, \end{array}$$

where *P* is the accumulated amount of methane produced per unit volatile organics at *t* moment (mL/gVS), *P*_max_ is the maximum production potential of methane (mL/gVS), *R*_max_ is the maximum production rate of methane (mL/gVS/d), *t* is the reaction period (d), and *λ* is the delay time (d).

The modified Gompertz equation is copied as follows:

(2) $$\begin{array}{c} M=P\times \exp\left\{\;-\exp\left[\;\frac{R_{m}\times e}{P}\left(\;\lambda -t\;\right)+1\;\right]\;\right\}, \end{array}$$

where *M* is the accumulated amount of methane produced per unit volatile organics at *t* moment (mL/gVS), *P* is the ultimate amount of methane produced per unit volatile organics (mL/gVS), *R*_*m*_ is the maximum production rate of methane (mL/gVS/d), *t* is the reaction period (d), and *λ* is the delay time (d).

The *P*, *P*_max_, and *P*_max_ values in the logistic equation are identical to the kinetic parameters *M*, *P*, and *R*_*m*_ in the modified Gompertz equation. This study adopted the nonlinear regression method using the Origin 8.0 software to carry out the kinetic parametric analysis for the logistic equation and modified Gompertz equation.

#### 2.4.2. First-Order Gas Production Model

A first-order gas production model [17] was developed based on previous studies demonstrating that biodegradable organics convert into methane in certain ratios [18]:

(3) $$\begin{array}{c} \frac{1}{t}\ln\left(\;\frac{dy_{t}}{dt}\;\right)=\frac{1}{t}\left(\;\ln\left(\;y_{m}\;\right)+\ln k\;\right)-k, \end{array}$$

where *y*_*m*_ is the theoretical amount of methane produced per unit volatile organics (mL/gVS), *y*_*t*_ is the practical amount of methane produced per unit volatile organics at *t* moment (mL/gVS), *t* is the reaction period (d), and *k* is the hydrolysis constant (d − 1).

In this way, ln⁡ (*y*_*m*_) + ln⁡*k* and *k* of the corresponding organics can be identified.

### 2.5. Initial Rate Method

The procedures of the initial rate method are as follows: Assuming the reaction follows *bB* + *cC* = *dA*, the reaction rate (*r*) can be obtained by

(4) $$\begin{array}{c} r=-kc{\left(\;B\;\right)}^{m}c{\left(\;C\;\right)}^{n}=kc{\left(\;A\;\right)}^{o}, \end{array}$$

where *c*(*B*) and *c*(*C*) are the initial concentrations of reactants *B* and *C*, while *c*(*A*) is the concentration of product *A* at the end of the reaction; *m*, *n*, and *o* represent the reaction orders of *B*, *C*, and *A*, respectively [16].

The initial rate method is based on different concentrations of reactants. In this study, the concentration of one reactant was assigned three different values for each group, while the concentrations of the other reactants remained constant. As the experiments proceeded, the concentrations of reactants and products were measured regularly.

Anaerobic fermentation refers to a process in which methane is produced from organics; therefore, the amount of methane produced can be recorded and used to investigate the hydrolysis constant and reaction order through the initial rate method. If *C*→*A* represents the conversion of VS to methane, then *r* = −*kc*^*n*^, where *c* denotes the concentration of the reactant, namely, the mass of VS at the beginning. Define the groups with 1, 2, and 3 times the initial TS concentrations as Groups *A*, *B*, and *C*, respectively. Assuming they show consistent hydrolysis constants, then *r*_1_ = −*kc*_1_^*n*^ and *r*_2_ = −*kc*_2_^*n*^, where *r*_1_/*r*_2_ can be replaced by *A*_1_/*A*_2_ (the ratio of gas produced at a specific moment); then, *A*_1_/*A*_2_ = *c*_1_^*n*^/*c*_2_^*n*^, which can be rearranged as *n* = (ln⁡(*A*_1_/*A*_2_))/(ln⁡(*c*_1_/*c*_2_)), from which *n* can be determined.

## 3. Results and Discussion

### 3.1. Entropy Change Analysis of Anaerobic Fermentation Process

Entropy is a state function used to describe and characterize the degree of chaos in a system. The entropy change of a process is only related to the system's initial state and final state, regardless of the approach or method. Δ*G* denotes the Gibbs free energy change, and Δ*G* = Δ*H* − *T*Δ*S*. Under conditions of constant temperature and pressure, the following associations are true: if Δ*S* > 0 and Δ*G* < 0, then a reaction spontaneously occurs; if Δ*S* < 0 and Δ*G* > 0, then a reaction occurs nonspontaneously; if Δ*S* = 0 and Δ*G* = 0, then the reaction is at an equilibrium state [19]. The entropy change analysis of the anaerobic fermentation process evaluates the process from a new perspective, providing a reliable scientific theory for the development and perfection of anaerobic fermentation technology, as well as the evaluation of treatment effects.

The complicated composition of kitchen waste makes the complete Δ*G* analysis of the fermentation process difficult; therefore, the digestion substrate of kitchen waste is simplified to glucose for convenience of analysis. In the process of anaerobic fermentation, glucose is first hydrolyzed and acidized into organic acids or alcohols with no less than 2*C*, then converted into acetic acid, H_2_, and CO_2_ by hydrogen-producing acetogens, and finally transformed to CH_4_ under the action of methanogens.  Table 1 elaborates the standard Gibbs free energy change when using glucose as the fermentation substrate and bacteria for hydrolysis, acid production, and fermentation [20, 21].

The data in Table 1 indicate that the standard Gibbs free energy changes for reactions in the hydrolysis, acid production, and fermentation phases are all smaller than zero, which implies that all the reactions take place spontaneously from left to right under standard conditions.

Therefore, the entropy values of these reactions are all greater than zero, and the processes increase entropy.

The standard Gibbs free energy change when using hydrogen-producing acetogens for the metabolism of organic acids and alcohols is shown in Table 2 [20, 22].

According to Table 2, the standard Gibbs free energy changes for most of the reactions at the hydrogen and acetic acid production phases are greater than zero, indicating that most of the reactions do not take place spontaneously from left to right under standard conditions. Therefore, the entropy of these phases is less than zero, indicating an entropy reduction process.

Furthermore, the numerical values of Δ*G*^*θ*^ are generally small. By appropriately modifying some of the reaction conditions, the energy change Δ*G*^*θ*^ can be adjusted to fall below 0, prompting the reactions that happen from left to right.

The standard Gibbs free energy change when using methanogens for the metabolism of intermediates is explained in Table 3 [20, 23].

The data in Table 3 show that the standard Gibbs free energy change values for reactions at the methane production phase are all less than zero, signifying that all of the reactions occur spontaneously from left to right under standard conditions. Therefore, the entropy in this phase is greater than zero, meaning that it is an entropy increasing process.

### 3.2. Result Discussion on Models for Anaerobic Digestion at Low TS Concentrations

#### 3.2.1. Result Discussion on Population Growth Model

The anaerobic fermentation process of kitchen waste with initial TS concentrations of 4%, 5%, and 6% was analyzed using a population growth model. Nonlinear fitting with the software Origin established the fitting parameters for the logistic equation and modified Gompertz equation describing the anaerobic fermentation of kitchen waste at different initial TS concentrations (see Tables 4 and 5).

Tables 4 and 5 reveal that although the values of *R*^2^ differ for different TS concentrations, they all fall between 0.95 and 1. This proves that the population growth model is suitable for simulating anaerobic fermentation and biogas production of kitchen waste at low TS concentrations. For different TS concentrations, the results also certify that the logistic equation and modified Gompertz equation are the right methods for the fitting process of anaerobic fermentation and biogas production of kitchen waste at various TS concentrations. In particular, the modified Gompertz equation shows the greatest gas production potential (540.94 mL/gVS) when applied to kitchen waste at 5% TS concentration, followed by the potentials for kitchen waste at 6% and 4% TS concentrations, which are 513.09 mL/gVS and 485.10 mL/gVS, respectively.

Because kitchen waste contains a great deal of readily decomposable organic starches like rice and steamed buns, as well as a moderate amount of organic protein like lean meat and eggs, the ratio between carbon and nitrogen during the anaerobic fermentation process is always appropriate. This not only accelerates the hydrolysis reaction but also benefits the growth and reproduction of microbes, thereby ensuring that the reaction proceeds smoothly. In this way, the experiment can generate biogas from the beginning, without any time delay.

#### 3.2.2. Result Discussion of First-Order Gas Production Model

Table 6 shows *k* and ln⁡ (*y*_*m*_) + ln⁡*k* at different TS concentrations, as predicted by the proposed first-order gas production model. The results show that *R*^2^ > 0.99 is valid for all initial TS concentrations, indicating good effectiveness on the part of the proposed model for anaerobic fermentation of kitchen waste at low TS concentrations. Hence, this model was used for theoretical analysis of experimentally obtained data.

*k* is an indicator of the proportion of biodegradable substances that have been digested, and large *k* values indicate high reaction rates. While *k* reflects the rate at which the methane is produced, *y*_*m*_ reflects the amount of gas produced. Therefore, ln⁡*y*_*m*_ + ln⁡*k* combines the overall amount of gas produced and the rate at which it is produced, making it a good indicator of the utilization of the biodegradable substances in the raw material. In other words, ln⁡*y*_*m*_ + ln⁡ *k* is a characteristic parameter of the gas production reaction. A large ln⁡*y*_*m*_ + ln⁡*k* value indicates high gas production capacity for the raw materials. Therefore, *k* values obtained in this study indicate the rates at which organic macromolecules were converted into compounds, while ln⁡*y*_*m*_ + ln⁡*k* values obtained in this study indicate the conversion efficiency of organic macromolecules into methane.

The results revealed that the hydrolysis constants corresponding to TS concentrations of 4%, 5%, and 6% were 0.2179, 0.1170, and 0.1430, respectively. Meanwhile, the values of ln⁡*y*_*m*_ + ln⁡*k* corresponding to TS concentrations of 4%, 5%, and 6% were 4.8109, 4.1292, and 4.2131, respectively, suggesting that the reaction rate was maximized at TS = 4% and minimized at TS = 5%. Additionally, *R*^2^ values of all the groups exceeded 0.99, demonstrating good efficacy of the proposed first-order gas production model in predicting anaerobic fermentation of kitchen waste.

According to the formulas and experimental data from the population growth model and first-order gas production model, both models achieve a satisfying fitting effect for the anaerobic fermentation and biogas production process of kitchen waste with low TS concentrations. In this study, the population growth model always yielded correlation coefficient *R*^2^ values between 0.95 and 1, while the first-order gas production model yielded correlation coefficient *R*^2^ values greater than 0.99. These results indicate that, for kitchen waste with low TS concentrations, the first-order gas production model has the best outcome in fitting the anaerobic fermentation and biogas production process, and it can therefore be used for the theoretical analysis of the experiments in general.

### 3.3. The Initial Rate Method for Anaerobic Digestion at Low TS Concentrations

**Figure 1:**
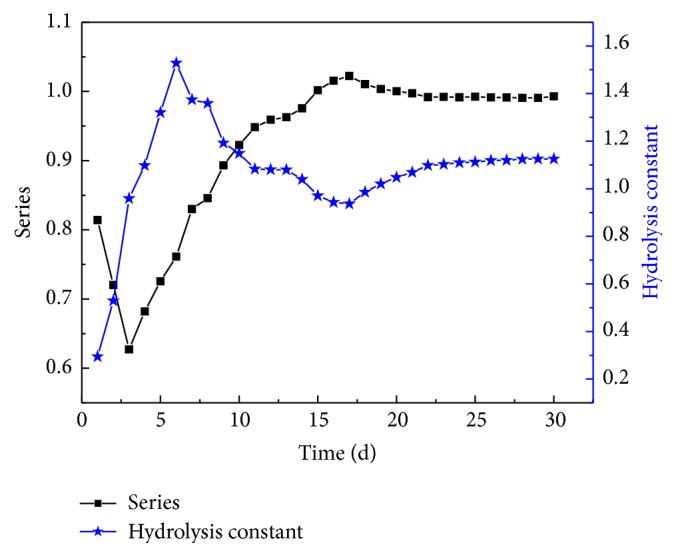
Hydrolysis constant and reaction order of anaerobic fermentation of kitchen waste at low TS concentrations.

Let the groups whose initial TS concentrations are 4%, 5%, and 6% be defined as Groups *A*, *B*, and *C*, respectively. Assuming they show consistent hydrolysis constants, then *r*_1_ = −*kc*_1_^*n*^, *r*_2_ = −*kc*_2_^*n*^, *n* = (ln⁡(*A*_1_/*A*_2_))/(ln⁡(*c*_1_/*c*_2_)). In this way, *n* can be obtained. During the tests, the hydrolysis constants and the reaction orders of all groups were measured daily, and the average values were recorded and shown in Figure 1.

The data indicates that the hydrolysis constant and reaction order exhibit opposite trends, although both stabilize eventually. The reaction order decreased during the first three days to a minimum at 0.6822, increased from Day 4 to Day 17, and finally decreased gradually until stabilizing at 0.99 from Day 24. The hydrolysis constant increased during the first six days, decreased from Day 7 to Day 17, and then increased steadily until stabilizing at 1.1252 from Day 24. Therefore, the hydrolysis of kitchen waste with initial TS concentrations of 4%, 5%, and 6% can be described by the first-order hydrolysis dynamic equations proposed.

### 3.4. The Initial Rate Method for Anaerobic Digestion at High TS Concentrations

In most studies concerning kitchen waste digestion, the first-order hydrolysis constant is obtained based on continuous dry fermentation. For instance, Wu et al. investigated anaerobic digestion of kitchen waste mixed with pig manure at mild temperatures [24]. In that study, feeding of organic matters increased gradually. Li et al. investigated the effects of loading rate on anaerobic digestion of kitchen waste during gradually increasing organic feeding [25]. In contrast to these studies, Lai et al. proposed a gas production model based on semicontinuous anaerobic digestion of kitchen waste mixed with pig manure during gradually increasing organic feeding [26]. Linke investigated the effects of organic loading rate on anaerobic digestion of tomato-based solid waste and obtained *k* based on first-order hydrolysis reactions [17]. Mähnert and Linke investigated the dynamics of first-order anaerobic digestion of energy crops mixed with animal manure and determined gas production both theoretically and practically, as well as identifying concentrations of volatile solids in the reactor, concentrations of outflow liquids, and the reaction rate constant [27]. However, none of these studies demonstrated the first-order model on the anaerobic digestion of kitchen waste alone. This study investigated the orders of sequenced reactions for waste with initial TS concentrations of 19%, 20%, and 21% using the initial rate method in order to provide reference for determining the reaction orders of dry fermentation of kitchen waste, as well as modelling continuous dry fermentation processes.

**Figure 2:**
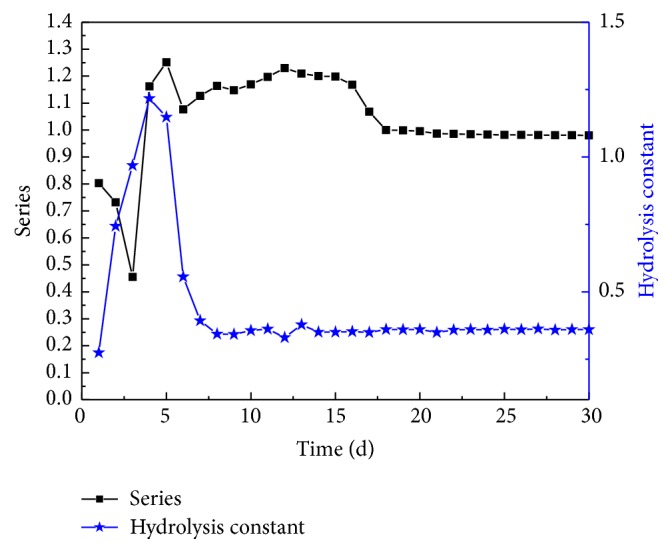
Hydrolysis constant and reaction order of anaerobic fermentation of kitchen waste at high TS concentrations.

The average hydrolysis constants and reaction orders for anaerobic digestion of kitchen waste with initial TS concentrations of 19%, 20%, and 21%, from the initial moment to a specific point, were obtained and shown in Figure 2.

As the data shows, the reaction order dropped from 0.8027 to 0.4552 during the first three days and then increased to 1.2511 on Day 5. Afterwards, the hydrolysis constant fluctuated and stabilized at 0.98 after Day 18. In contrast, the hydrolysis constant increased during the first four days until it reached 1.1479; then, it decreased from Day 5 to Day 8, increased again, and stabilized at 0.3507 after Day 14. These results suggest that hydrolysis of kitchen waste with initial TS concentrations of 19%, 20%, and 21% can be described by the first-order hydrolysis dynamic equations proposed.

## 4. Conclusions

(1) This paper analyzed the entropy changes corresponding to each phase of the anaerobic biological treatment process. In the hydrolysis and acidification phases, the standard Gibbs free energy change Δ*G*^*θ*^ < 0, and the reactions happen spontaneously; therefore, it is an entropy increasing process. In contrast, in the hydrogen and acetic acid production phases, Δ*G*^*θ*^ > 0 for most of the reactions; therefore, it is an entropy reduction process. In the methane production phase, Δ*G*^*θ*^ < 0, and the reactions take place spontaneously; therefore, it is an entropy increasing process. Most of the reactions are spontaneous during the anaerobic biological treatment process; only the hydrogen and acetic acid production phases are nonspontaneous. From the perspective of thermodynamics, these phases require additional energy and matter supply.

(2) This study determined the hydrolysis constants and reaction orders for anaerobic digestion of kitchen waste using the initial rate method, in addition to examining the population growth model and first-order hydrolysis model. The results prove that the first-order hydrolysis model can better reflect the kinetic process of gas production. The results demonstrate the application of the proposed first-order gas production model in anaerobic digestion of kitchen waste.

(3) During 30 days of anaerobic digestion of kitchen waste with low TS concentrations, the hydrolysis constants and the reaction orders demonstrated opposite trends, and both stabilized after 24 days at 0.99 and 1.1252, respectively.

(4) During 30 days of anaerobic digestion of kitchen wastes with high TS concentrations, the hydrolysis constants and reaction orders stabilized at 0.98 (after 18 days) and 0.3507 (after 14 days), respectively. These results demonstrate that hydrolysis of kitchen waste with both low TS concentrations and high TS concentrations can be described using the proposed first-order hydrolysis dynamic equations.

(5) Given sufficient reaction time, the hydrolysis involved in anaerobic fermentation of kitchen waste can be regarded as a first-order reaction in terms of reaction kinetics.

## Figures and Tables

**Table 1:** Standard Gibbs free energy change when using glucose as fermentation substrate and bacteria for hydrolysis, acid production, and fermentation.

Reaction equation (pH = 7, *T* = 298.15 K)	Δ*G*^*θ*^ (kJ/mol)	Δ*S*
C_6_H_12_O_6_ + 4H_2_O + 2NAD^+^⟶ 2CH_3_COO^−^ + 2HCO_3_^−^ + 2NADH + 2H_2_ + 6H^+^	−215.67 < 0	>0
C_6_H_12_O_6_ + 2NADH ⟶ 2CH_3_CH_2_COO^−^ + 2H_2_O + 2NAD^+^	−357.87 < 0	>0
C_6_H_12_O_6_ + 4H_2_O ⟶ 2CH_3_COO^−^ + 2HCO_3_^−^ + 4H_2_ + 4H^+^	−184.20 < 0	>0
C_6_H_12_O_6_ + 2H_2_O ⟶ CH_3_CH_2_CH_2_COO^−^ + 2HCO_3_^−^ + 2H_2_ + 3H^+^	−261.46 < 0	>0
C_6_H_12_O_6_ + 2H_2_O + 2NADH ⟶ 2CH_3_CH_2_OH + 2HCO_3_^−^ + 2NAD^+^ + 2H_2_	−234.83 < 0	>0
C_6_H_12_O_6_⟶ 2CH_3_CHOHCOO^−^ + 2H^+^	−217.70 < 0	>0

**Table 2:** Standard Gibbs free energy change when using hydrogen-producing acetogens for metabolism of organic acids and alcohols.

Reaction equation (pH = 7, *T* = 298.15 K)	Δ*G*^*θ*^ (kJ/mol)	Δ*S*
CH_3_CH_2_OH + H_2_O ⟶ CH_3_COO^−^ + 2H_2_ + 2H^+^	+9.6 > 0	<0
CH_3_CH_2_COO^−^ + 3H_2_O ⟶ CH_3_COO^−^ + HCO_3_^−^ + H^+^ + 3H_2_	+76.1 > 0	<0
CH_3_CH_2_COO^−^ + 2HCO_3_^−^⟶ CH_3_COO^−^ + H^+^ + 3HCOO^−^	+72.4 > 0	<0
CH_3_CH_2_CH_2_COO^−^ + 2H_2_O ⟶ 2CH_3_COO^−^ + H + 2H_2_	+48.1 > 0	<0
CH_3_CH_2_CH_2_COO^−^ + 2HCO_3_^−^⟶ 2CH3COO^−^ + H^+^ + 2HCOO^−^	+45.5 > 0	<0
CH_3_CH_2_CH_2_CH_2_COO^−^ + 2H_2_O ⟶ CH_3_COO^−^ + CH_3_CH_2_COO^−^ + H^+^ + 2H_2_	+25.1 > 0	<0
CH_3_CHOHCOO^−^ + 2H_2_O ⟶ CH_3_COO^−^ + HCO_3_^−^ + H^+^ + 2H_2_	−4.2 < 0	>0

**Table 3:** Standard Gibbs free energy change when using methanogens for metabolism of intermediates.

Reaction equation (pH = 7, *T* = 298.15 K)	Δ*G*^*θ*^ (kJ/mol)	Δ*S*
4CH_3_CH_2_COO^−^ + 3H_2_O ⟶ 4CH_3_COO^−^ + HCO_3_^−^ + H^+^ + 3CH_4_	−102.0 < 0	>0
2CH_3_CH_2_CH_2_COO^−^ + HCO_3_^−^ + H_2_O ⟶ 4CH_3_COO^−^ + H^+^ + CH_4_	−39.4 < 0	>0
CH_3_COOH ⟶ CO_2_ + CH_4_	−31.0 < 0	>0
4HCOOH ⟶ 3CO_2_ + 2H_2_O + CH_4_	−130.1 < 0	>0
4H_2_ + HCO_3_^−^ + H^+^⟶ 3H_2_O + CH_4_	−135.6 < 0	>0
2CH_3_CH_2_OH + CO_2_⟶ 2CH_2_COOH + CH_4_	−116.3 < 0	>0
CH_3_OH + H_2_⟶ H_2_O + CH_4_	−112.5 < 0	>0
4CH_3_OH ⟶ CO_2_ + 2H_2_O + 3CH_4_	−104.9 < 0	>0
4CH_3_NH_2_ + 2H_2_O ⟶ CO_2_ + 4NH_3_ + 3CH_4_	−75.0 < 0	>0
2(CH_3_)_2_NH + 2H_2_O ⟶ CO_2_ + 2NH_3_ + 3CH_4_	−73.2 < 0	>0
4(CH_3_)_3_N + 6H_2_O ⟶ 3CO_2_ + 4NH_3_ + 9CH_4_	−74.3 < 0	>0
2(CH_3_)_2_S + 2H_2_O ⟶ CO_2_ + 2H_2_S + 3CH_4_	−73.8 < 0	>0

**Table 4:** Fitting parameters for logistic equation.

TS/%	*P*_max_	*R*_max_	*λ* (d)	*R* ^2^
	(mL/gVS)	(mL/gVS/d)		
4	480.60	21.91	−7.42	0.95125
5	534.81	42.48	−1.18	0.97202
6	503.78	32.24	−1.17	0.99414

**Table 5:** Fitting parameters for modified Gompertz equation.

TS (%)	*P*	*R*_*m*_	*λ* (d)	*R* ^2^
	(mL/gVS)	(mL/gVS/d)		
4	485.10	26.52	−5.53	0.95981
5	540.94	32.18	−4.95	0.98597
6	513.09	23.67	−6.34	0.99705

**Table 6:** Parameters of anaerobic fermentation of kitchen waste at different TS concentrations predicted by the proposed first-order gas production model.

Initial TS concentration	Parameter		
	ln⁡(*y*_*m*_) + ln⁡*k*	*k*	*R* ^2^
4%	4.8109	0.2179	0.9930
5%	4.1292	0.1170	0.9938
6%	4.2131	0.1430	0.9965

## References

[1] Xie W. P., Liang Y. J., He D. W. (2008). Food waste of resources technology status and progress. *Environmental Sanitation Engineering*.

[2] Yin C.-H., Dong X., Lv L. (2013). Economic production of probiotics from kitchen waste. *Food Science and Biotechnology*.

[3] Ribbens S., Dewulf J., Koenen F. (2008). A survey on biosecurity and management practices in Belgian pig herds. *Preventive Veterinary Medicine*.

[4] Yang S. Y., Ji K. S., Baik Y. H., Kwak W. S., McCaskey T. A. (2006). Lactic acid fermentation of food waste for swine feed. *Bioresource Technology*.

[5] Manfredi S., Pant R. (2013). Improving the environmental performance of bio-waste management with life cycle thinking (LCT) and life cycle assessment (LCA). *International Journal of Life Cycle Assessment*.

[6] Hanc A., Szakova J., Svehla P. (2012). Effect of composting on the mobility of arsenic, chromium and nickel contained in kitchen and garden waste. *Bioresource Technology*.

[7] den Boer E., den Boer J., Jaroszyńska J., Szpadt R. (2012). Monitoring of municipal waste generated in the city of Warsaw. *Waste Management and Research*.

[8] Liu G., Liu X., Li Y., He Y., Zhang R. (2011). Influence of pH adjustment and inoculum on anaerobic digestion of kitchen waste for biogas producing. *Journal of Biobased Materials and Bioenergy*.

[9] Tauseef S. M., Abbasi T., Abbasi S. A. (2013). Energy recovery from wastewaters with high-rate anaerobic digesters. *Renewable and Sustainable Energy Reviews*.

[10] Nasir I. M., Ghazi T. I. M., Omar R. (2012). Production of biogas from solid organic wastes through anaerobic digestion: a review. *Applied Microbiology and Biotechnology*.

[11] Wang A. J., Li W. W., Yu H. Q. (2012). Advances in biogas technology. *Advances in Biochemical Engineering Biotechnology*.

[12] Song C. H., Wei Z. M., Xi B. D. (2013). Effect of heavy metals biogas mixed material composting. *Safety and Environment*.

[13] Liu Z. Q. (2012). *Optimize Microalgae Culture, Harvesting and Cultivation of Microalgae Biogas Research*.

[14] Chen J. Y. Determination of the reaction order with respect to. *Chemistry of University*.

[15] Yan X., Zhang Y. H., Li Z. (2012). Experience teaching method for determining the reaction order. *Xinjiang Normal University*.

[16] Wanasolo W., Manyele S. V., Makunza J. (2013). A kinetic study of anaerobic biodegradation of food and fruit residues during biogas generation using initial rate method. *Engineering*.

[17] Linke B. (2006). Kinetic study of thermophilic anaerobic digestion of solid wastes from potato processing. *Biomass and Bioenergy*.

[18] Feng L., Kou H. L., Kou W. (2015). A hydrolysis of food waste and its components dynamics model. *Environmental Pollution and Control*.

[19] Department of Inorganic Chemistry Dalian University of Technology (2006). *Inorganic Chemistry*.

[20] Ren N.-Q., Wang A.-J., Ma F. (2005). *Physiological Ecology of Acidogenic Fermention Microbial*.

[21] Han S.-K., Shin H.-S. (2004). Performance of an innovative two-stage process converting food waste to hydrogen and methane. *Journal of the Air and Waste Management Association*.

[22] Wang Q., Kuninobu M., Ogawa H. I., Kato Y. (1999). Degradation of volatile fatty acids in highly efficient anaerobic digestion. *Biomass and Bioenergy*.

[23] Van Lier J. B., Grolle K. C., Frijters C. T., Stams A. J., Lettinga G. (1993). Effects of acetate, propionate, and butyrate on the thermophilic anaerobic degradation of propionate by methanogenic sludge and defined cultures. *Applied and Environmental Microbiology*.

[24] Wu Y., Zhang W. Y., Pang Y. (2011). Semi-continuous mixing food waste and manure anaerobic fermentation kinetics of. *Anhui Agricultural Sciences*.

[25] Li L. Q., Li X. J., Zheng M. X. (2009). Tomato waste semi-continuous anaerobic fermentation experiment and kinetic model. *China Biogas*.

[26] Lai X., Zhang W., Zhang L., Chen J. (2015). Prediction of gas production of semi-continuous anaerobic co-digestion based on artificial neural network. *Chinese Journal of Environmental Engineering*.

[27] Mähnert P., Linke B. (2009). Kinetic study of biogas production from energy crops and animal waste slurry: effect of organic loading rate and reactor size. *Environmental Technology*.

